# Development of Recombinant Multi-Epitope Hepatitis C Virus (HCV) Protein for Hepatitis C Diagnosis

**DOI:** 10.3390/v18101049

**Published:** 2026-09-22

**Authors:** Raíssa Martins Deodato, Mayara Torquato Lima da Silva, Diego Allonso, Erik Machado Ferreira, Julia Trece Marques Possas, Jurema Corrêa da Mota, Livia Melo Villar

**Affiliations:** 1 Viral Hepatitis Laboratory, Oswaldo Cruz Institute, Rio de Janeiro 21040-900, Brazil; juhtrece@gmail.com (J.T.M.P.); correamota@gmail.com (J.C.d.M.); 2 Laboratory of Biotechnology and Structural Bioengineering, Federal University of Rio de Janeiro, Rio de Janeiro 21941-901, Brazil; mayara.torquato@gmail.com (M.T.L.d.S.); diegoallonso@gmail.com (D.A.); 3 Recombinant Technology Laboratory, Institute of Technology on Immunobiologicals, Rio de Janeiro 21040-900, Brazil; erik.ferreira@bio.fiocruz.br

**Keywords:** recombinant protein, diagnostic immunoassay, hepatitis C, serodiagnosis

## Abstract

Hepatitis C virus (HCV) infection poses a major challenge to global public health, highlighting the need for accessible and reliable diagnostic tools to support large-scale screening and early detection. In this study, a recombinant multi-epitope HCV protein was designed, expressed, and evaluated as a diagnostic antigen for the detection of anti-HCV antibodies. The synthetic gene was constructed using immunodominant regions (core, NS3, NS4, NS5A, and NS5B) representative of HCV genotypes 1a, 1b, and 3a. The recombinant protein was expressed in *Escherichia coli* and purified by affinity chromatography, yielding a soluble protein with an approximate molecular weight of 43 kDa and antigenicity confirmed by Western blot analysis. An enzyme-linked immunosorbent assay (ELISA) was developed in-house using the recombinant protein as the capture antibody. Diagnostic performance was evaluated using a panel of 260 human serum samples, including 127 anti-HCV reactive and 133 anti-HCV non-reactive specimens, and receiver operating characteristic (ROC) curve analysis. The assay demonstrated excellent performance with an area under the curve (AUC) of 0.968, sensitivity of 86.6%, specificity of 98.5%, and overall accuracy of 92.7%. Diagnostic performance was evaluated using receiver operating characteristic (ROC) curve analysis. The assay demonstrated excellent performance in serum samples, with an area under the curve (AUC) of 0.968, a sensitivity of 86.6%, and a specificity of 98.5%. These results demonstrate that the recombinant multi-epitope antigen represents a robust and scalable biomolecular platform for HCV serodiagnosis, contributing to strategies that expand access to hepatitis C screening.

## 1. Introduction

The hepatitis C virus (HCV) belongs to the Flaviviridae family and the Hepacivirus genus. It is an enveloped virus with single-stranded, positive-sense RNA, whose genome is approximately 9.6 kb [1]. Infection occurs primarily through contact with contaminated blood, with vertical and sexual transmission being less common [2,3]. Its E1 and E2 glycoproteins, located on the viral envelope, are essential for viral entry into hepatocytes [4]. Non-structural proteins include NS3, NS4, NS5A, and NS5B, which are crucial for viral replication [2].

Despite advances in the development of direct-acting antivirals (DAAs), which can cure more than 95% of infected individuals [5,6], hepatitis C remains a significant cause of chronic liver disease and mortality worldwide. According to the World Health Organization (WHO), approximately 50 million people are living with chronic HCV infection globally, with about 1.0 million new infections occurring each year [3]. In 2022 alone, an estimated 242,000 deaths were attributed to hepatitis C, mainly due to complications such as cirrhosis and hepatocellular carcinoma. In Brazil, 342,328 confirmed cases of hepatitis C were reported between 2000 and 2024, reflecting the continued public health burden of the disease in the country [7]. Although highly effective treatment is available, access to diagnosis and treatment remains limited in many settings, representing a major barrier to disease control.

Currently, there is no effective vaccine against HCV, and most acute infections are asymptomatic, contributing to delayed diagnosis and ongoing transmission. Because new infections are frequently asymptomatic, many individuals remain undiagnosed until advanced liver disease develops, reinforcing the critical importance of early detection strategies [3].

In response to this global health challenge, the WHO established targets to eliminate viral hepatitis as a public health threat by 2030, aiming to reduce new infections by 90% and mortality by 65%. Achieving these goals requires scaling up prevention, testing, and treatment services, particularly among populations at higher risk of infection. Expanding access to simple, reliable, and decentralized diagnostic tools is therefore considered a key priority within the Global Health Sector Strategies (GHSS) for the period 2022–2030 [3].

Laboratory diagnosis of HCV infection is performed through a two-step process. First, serological assays detect anti-HCV antibodies to identify individuals previously exposed to the virus. If the antibody test is positive, a nucleic acid test (NAT) for HCV RNA is required to confirm active infection and determine the need for treatment. Serological assays such as the enzyme-linked immunosorbent assay (ELISA) are widely used, highly reliable, and considered reference methods for large-scale screening programs. Their scalability, relatively low cost, and compatibility with different antigen formats make ELISA-based platforms particularly suitable for diagnostic innovation [8,9]. Commercial anti-HCV immunoassays are based on recombinant antigens and exhibit excellent diagnostic performance. Nevertheless, the continuous development of recombinant antigens remains an active area of research, driven by advances in protein engineering that enable the rational design of antigens with broader epitope representation and improved standardization. In this context, recombinant multi-epitope proteins have emerged as an attractive strategy because they combine immunodominant regions from different viral proteins into a single construct. This approach seeks to maximize antibody recognition while simplifying antigen production, providing a standardized recombinant molecule that can be incorporated into serological assays. Furthermore, the inclusion of epitopes from different viral proteins and genotypes may contribute to broad antibody recognition across genetically diverse HCV strains and different stages of infection [10]. These characteristics make multi-epitope recombinant antigens promising candidates for the development of robust and scalable serological assays, particularly for in-house diagnostic platforms [11,12].

The objective of this study was to develop and evaluate an in-house ELISA assay using a recombinant HCV protein with multiple epitopes for the detection of anti-HCV antibodies in serum samples. Unlike commercially available assays, this study focuses on the development and validation of a single recombinant multi-epitope antigen designed to incorporate immunodominant regions from different HCV proteins and genotypes into an in-house ELISA platform. By evaluating its diagnostic performance, we aimed to determine whether this strategy could provide a standardized, scalable, and cost-effective alternative for serological screening while maintaining high diagnostic accuracy. This strategy aims to expand diagnostic coverage, improve test accuracy, and contribute to global efforts to achieve the WHO’s goals for the elimination of hepatitis by 2030.

## 2. Materials and Methods

### 2.1. Design and Cloning of the Synthetic Gene

The synthetic gene was constructed from immunogenic peptides derived from hepatitis C virus (HCV) proteins: core, NS3, NS4, NS5A, and NS5B, representative of genotypes 1a, 1b, and 3a, which are the most prevalent in Brazil (Table 1). The regions were connected by flexible linkers, and the gene was inserted into the pET21a expression vector, chemically synthesized by GenScript Corp—Piscataway, NJ, USA, with codon optimization for expression in *Escherichia coli* (Figure 1). The insert was flanked by the restriction enzymes XhoI and BamHI, enabling the expression of the recombinant protein with a histidine tail to facilitate its subsequent purification.

The design of the recombinant multiepitope protein followed a sequential workflow. Initially, the HCV proteins Core, NS3, NS4, NS5A, and NS5B were selected based on published evidence demonstrating their immunogenicity and relevance for serological diagnosis [13]. Genotypes 1a, 1b, and 3a were selected because they represent the most prevalent HCV subtypes circulating in Brazil. The corresponding polyprotein sequences (UniProt: P27958, P26663, and Q81495) were analyzed using three independent B-cell epitope prediction tools: IEDB B Cell Epitope Prediction (Method BepiPred-2.0: Se-quencial B-Cell Epitope Predictor), Predicting Antigenic Peptides, and SVMTriP. The predicted epitopes were mapped onto their respective protein sequences, and regions consistently identified by at least two prediction algorithms were considered immu-nodominant and selected for further analysis. Multiple sequence alignments of the se-lected regions from the three HCV genotypes were then performed to generate con-sensus sequences, which were incorporated into the synthetic gene construct. The se-lected antigenic regions were connected using flexible Gly-Gly-Gly-Gly-Ser (GGGGS) linkers to minimize steric hindrance between adjacent epitopes, facilitate independent folding, and preserve epitope accessibility within the recombinant protein. The result-ing multiepitope sequence was subsequently evaluated using VaxiJen 2.0 with a threshold of 0.4 to confirm its predicted antigenicity.

### 2.2. Expression and Purification of Recombinant Protein

The recombinant multi-epitope protein was expressed in *E. coli* BL21 Star (DE3) cells (Thermo Scientific^®^, Waltham, MA, USA), which are widely used for the large-scale expression of non-toxic recombinant proteins. Cell transformation was performed using the heat shock method, followed by selection of transformed clones under antibiotic selective pressure. Glycerol stocks were prepared by mixing 800 μL of liquid culture with 200 μL of 80% sterile glycerol and stored at −80 °C for subsequent experiments.

To determine the optimal conditions for recombinant protein expression, induction assays were performed using BL21 Star (DE3) cells transformed under different parameters, including temperature (25 °C and 37 °C), induction time (2, 4, and 18 h), and IPTG concentration (0.5 mM, 1.0 mM, and 1.5 mM). The highest expression levels were observed at 37 °C for 18 h, using IPTG concentrations of 0.5 mM or 1.0 mM.

Protein solubility was assessed after cell lysis by sonication. No inclusion bodies were observed, and the fusion protein was detected predominantly in the soluble fraction of the cell extract.

The bacterial pellet was resuspended in extraction buffer (8 M urea, 50 mM NaH_2_PO_4_, 300 mM NaCl, 10 mM imidazole, pH 8.0), and protein extraction and purification were performed under denaturing conditions. The cells were mechanically lysed using an ultrasonic processor. The lysate was then centrifuged at 9000 rpm for 20 min at 4 °C, and the supernatant was collected as the soluble fraction containing the target protein.

Recombinant protein purification was carried out by immobilized metal affinity chromatography (IMAC) using HisPur™ Ni-NTA Resin (Thermo Scientific, Waltham, MA, USA) mounted on an ÄKTA chromatography system. The resin was pre-equilibrated with extraction buffer, and purification was based on the specific interaction between the polyhistidine tag (His-tag) fused to the recombinant protein and nickel ions immobilized on the chromatographic matrix. After sample loading, the column was washed with buffer containing 4 M urea, 50 mM NaH_2_PO_4_, 300 mM NaCl, and 20 mM imidazole (pH 8.0) to remove non-specifically bound proteins. The target protein was subsequently eluted using elution buffer (0 mM NaH_2_PO_4_, 300 mM NaCl, and 500 mM imidazole (pH 8.0). Purified fractions were analyzed by 12% SDS-PAGE, spectrophotometry at 280 nm, and Western blotting.

### 2.3. Gel Electrophoresis and Western Blotting

The expression of the recombinant protein was checked by 12% SDS-PAGE. The samples were prepared in a sample buffer containing glycerol, SDS, Tris-HCl, Coomassie blue, and β-mercaptoethanol and applied to the gel wells together with a molecular weight marker (Kaleidoscope, Bio-Rad Laboratories, Hercules, CA, USA), and electrophoresis was performed at 150 volts and 25 milliamps for approximately one hour. After electrophoresis, the gels were stained with Coomassie blue solution.

For the Western blot step, the gels were loaded with the serum samples and the Precision Plus Protein™ Dual Color Standard (Bio-Rad Laboratories, Hercules, CA, USA) as the molecular weight marker and transferred to nitrocellulose membranes (0.22 or 0.45 µm) at 25 volts for about 30 min using a trans-blot system (Bio-Rad). After the transfer, the membrane was blocked for 1 h in a 5% skim milk solution prepared in 0.05% TBST, under constant shaking. Then, the membrane was incubated overnight with the primary antibody, which consisted of serum samples from patients reactive and non-reactive to anti-HCV antibodies, diluted 1:10 in TBST containing milk. The incubation with the anti-human secondary antibody conjugated to peroxidase (Sigma-Aldrich, St. Louis, MO, USA), also diluted 1:5.000 in TBST with milk, was carried out for 1 h. Immunoreactive bands were visualized using a chemiluminescent substrate and detected with an ImageQuant LAS 500 imaging system (GE Healthcare Bio-Sciences AB, Uppsala, Sweden).

### 2.4. Study Population and Sample Collection

A total of 260 biological samples from the Viral Hepatitis Laboratory biobank were included in this study and characterized using reference methodologies. The samples were obtained from adult individuals (≥18 years) attending the Viral Hepatitis Outpatient Clinic at Fiocruz for clinical evaluation and management. All participants voluntarily agreed to participate in the study, provided written informed consent, and donated blood and saliva samples for research purposes in accordance with the approved study protocol. The exclusion criteria were refusal to provide informed consent or biological samples unsuitable for laboratory analysis.

The sample was selected by convenience and consisted of participants who met the eligibility criteria and agreed to participate during the data collection period. Therefore, this was a non-probability sample, which does not allow inferences to the target population of patients receiving care at the outpatient clinic.

Whole blood samples had previously been collected by venipuncture into tubes containing an anticoagulant and centrifuged at 1500 rpm for 10–15 min. The resulting supernatant was carefully aspirated and stored under controlled conditions until analysis.

Serological screening for anti-HCV antibodies was performed using electrochemiluminescence immunoassay (ECLIA). The anti-HCV ECLIA result was considered the reference standard for evaluating the diagnostic performance of the in-house ELISA, as both assays are designed to detect anti-HCV antibodies. Reactive samples were further tested for HCV RNA by automated reverse transcription polymerase chain reaction (RT-PCR) using the Abbott RealTime HCV^®^ (Abbott Molecular Inc., Des Plaines, IL, USA) assay exclusively to confirm active infection.

Among the 260 serum samples analyzed, 127 were anti-HCV reactive, and 133 were anti-HCV non-reactive. The mean age of the anti-HCV-reactive and non-reactive groups was 56.1 ± 12.1 and 50.0 ± 11.7 years, respectively, with males comprising 39.4% and 45.1% of each group. HCV RNA was detected in 58 (45.7%) of the anti-HCV-reactive samples, with a mean viral load of 5.9 ± 0.9 log10 IU/mL (Table 2). A serum panel comprising comparable numbers of anti-HCV-reactive and non-reactive samples was assembled to evaluate the diagnostic performance of the recombinant antigen. The inclusion of both reactive and non-reactive specimens enabled the assessment of sensitivity, specificity, overall accuracy, ROC curve performance, and agreement with the reference method. HCV RNA testing was performed solely to characterize active infection among anti-HCV-reactive individuals and was not used as the reference standard. Because the in-house ELISA was designed to detect anti-HCV antibodies rather than active infection, all diagnostic performance analyses were performed using the anti-HCV ECLIA results as the reference standard.

### 2.5. In-House Enzyme-Linked Immunosorbent Assay (ELISA)

After protein purification, the in-house ELISA assay was standardized using serum samples and a multi-epitopic recombinant protein as the capture antigen. Initially, different concentrations of the recombinant protein (2.5, 5, 10, and 50 µg/mL) were evaluated to determine the optimal amount of antigen capable of providing adequate discrimination between positive and negative samples. For this purpose, flat-bottom polystyrene microplates (Nunc^®^, Thermo Fisher Scientific, Roskilde, Denmark) were coated with 100 µL of recombinant protein diluted in PBS containing 0.05% bovine serum albumin (PBS-BSA) and incubated overnight at 4 °C. A coating concentration of 10 µg/mL was selected for subsequent experiments, as it provided the best discrimination between anti-HCV-reactive and non-reactive samples.

The need for a blocking step was also evaluated during assay optimization, and blocking with 1% PBS-BSA was selected to minimize nonspecific binding. After this step, the plates were washed with PBS containing 0.5% Tween 20 (PBST).

Subsequently, different dilutions of the serum samples (1:1, 1:10, 1:50, and 1:100) in 0.05% PBS-BSA were tested by adding 100 µL per well, followed by incubation for 1 h at 37 °C. A serum dilution of 1:10 was selected for the optimized assay protocol. After a further wash with PBST, peroxidase-labeled anti-human conjugate (Sigma-Aldrich) was evaluated at dilutions of 1:30,000 and 1:60,000 in 0.05% PBS-BSA. The 1:60,000 dilutions were selected for the final protocol. The optimal assay conditions were selected based on the highest positive-to-negative optical density ratio while maintaining low background reactivity among non-reactive samples. The conjugate was added (100 µL/well) and incubated for 1 h at 37 °C.

After an additional washing step, the enzymatic reaction was developed with 100 µL of tetramethylbenzidine (TMB) (EMD Millipore, Burlington, MA, USA) for 10 min at 37 °C. The reaction was stopped by adding 50 µL of 1N sulfuric acid, and the absorbance was measured at 450 nm in a microplate spectrophotometer, using 630 nm as the reference wavelength.

### 2.6. Statistical Analysis

The dataset included two primary variables: (i) the anti-HCV result obtained by the reference electrochemiluminescence immunoassay (ECLIA), which was used as the reference.

Diagnostic performance was evaluated by Receiver Operating Characteristic (ROC) curve analysis. ROC curves were constructed using the reference test result as the dependent variable and the corresponding OD values as the predictor variable.

The area under the ROC curve (AUC) was calculated as a measure of the overall discriminative ability of the assay, together with its corresponding 95% confidence interval (95% CI).

The optimal cut-off value was determined using the Youden Index (J = sensitivity + specificity − 1), maximizing the combined sensitivity and specificity. Based on the selected cut-off, the following diagnostic performance parameters were calculated: sensitivity, specificity, and overall accuracy, along with their respective 95% confidence intervals.

In addition, agreement between the in-house ELISA and the reference methods for anti-HCV antibody detection was assessed using Cohen’s kappa coefficient (κ), based on the classification of samples as positive or negative according to the established cut-off. Overall concordance (%) was calculated, and the κ coefficient was used to estimate the level of agreement beyond that expected by chance, with their respective 95% confidence intervals. Kappa values were interpreted according to the Landis and Koch classification as follows: poor (<0.00), slight (0.00–0.20), fair (0.21–0.40), moderate (0.41–0.60), substantial (0.61–0.80), and almost perfect (0.81–1.00).

The limit of detection (LOD) of the in-house ELISA was evaluated using tenfold serial dilutions of an anti-HCV-positive serum sample prepared in normal human serum confirmed to be negative for anti-HCV antibodies. The starting positive sample showed high reactivity in the assay, with an OD value above 3.0, and was confirmed to have an HCV RNA viral load of 5 log_10_ IU/mL. All dilution points were subsequently processed according to the previously established ELISA protocol, including the sample dilution required for application to the ELISA plate. The LOD was defined as the highest dilution of the anti-HCV-positive sample that produced a mean OD value equal to or greater than the previously established cut-off of 0.405. Dilutions yielding mean OD values below 0.405 were considered non-reactive.

Batch-to-batch variation was evaluated using two independent production batches of the recombinant multiepitope HCV protein. For this analysis, five anti-HCV-positive and five anti-HCV-negative serum samples were tested in triplicate for each protein batch under the same ELISA conditions. The OD value for each sample was calculated as the mean of three technical replicates. Batch-to-batch variation was assessed by comparing the mean OD values obtained for each sample between the two independent batches. The coefficient of variation (CV%) was calculated for each group, and statistical comparisons between batches were performed using the paired Wilcoxon test.

The repeatability and reproducibility of the in-house ELISA were assessed using negative controls (NC) and positive controls (PC). Each control was evaluated by two independent operators over three experimental days, with three replicate measurements performed by each operator on each day [14,15].

For the assessment of inter-operator reproducibility, the three replicate measurements obtained by each operator on each experimental day were summarized, resulting in nine measurements per operator for each control. The median and interquartile range (IQR; Q1–Q3) were calculated separately for NC and PC samples. The distribution of the measurements obtained by each operator was visualized using boxplots.

Differences between operators were assessed using the Wilcoxon signed-rank test for paired samples. A *p*-value < 0.05 was considered statistically significant.

All statistical analyses were performed using the open-source R programming language, version 4.4.1 (R Core Team, 2026—Vienna, Austria), with the dplyr, ggplot2, flextable, and officer packages.

## 3. Results

### 3.1. Expression and Characterization of the Recombinant Protein

Prior to experimental characterization, in silico antigenicity prediction using VaxiJen v2.0 classified the recombinant multi-epitope HCV protein as probable antigenic, with a score of 0.5113, exceeding the threshold value of 0.4 for viral proteins. The recombinant protein was subsequently successfully expressed in *E. coli.* SDS-PAGE analysis revealed a predominant protein band with an approximate molecular weight of ~43 kDa, consistent with the predicted size of the construction (Figure 2).

Following affinity chromatography (Ni-NTA), the target protein was obtained with high purity, as evidenced by the enrichment of the corresponding band in the eluted fractions. Spectrophotometric analysis at 280 nm indicated protein concentrations exceeding 1 mg/mL in the collected fractions.

Western blot analysis confirmed the antigenicity of the recombinant protein, showing specific reactivity with anti-HCV-positive serum compared to negative control samples (Figure 3).

### 3.2. Human Anti-Hepatitis C Antibodies Recognize Multiepitope HCV

The optimized in-house ELISA protocol employed a coating concentration of 10 µg/mL of the recombinant protein was found to be sufficient to generate an adequate signal, yielding a mean positive optical density (OD) of 0.836 ± 0.099 and a ΔOD of 0.564 between positive and negative controls. Based on these results, this concentration was selected for subsequent experiments. Following the blocking step, anti-HCV-positive and anti-HCV-negative serum samples diluted 1:10 in PBS-BSA were added to the wells (100 µL/well).

To evaluate the diagnostic performance of the recombinant antigen, a total of 260 human serum samples were analyzed in triplicate, including 127 anti-HCV-positive and 133 anti-HCV-negative specimens. The assay demonstrated excellent diagnostic performance, with an area under the curve (AUC) of 0.968 (95% CI: 0.947–0.989). The optimal cut-off value, determined using the Youden index, was 0.405 OD, resulting in a sensitivity of 86.6%, specificity of 98.5%, and overall accuracy of 92.7% (Figure 4).

Agreement between the in-house ELISA and the reference anti-HCV ECLIA assay was also evaluated. The assay showed an overall concordance rate of 92.69%, with a Cohen’s kappa coefficient of 0.853, indicating almost perfect agreement between the methods according to the Landis and Koch classification (Table 3).

The analytical sensitivity of the in-house ELISA was evaluated by testing serial dilutions of an anti-HCV-positive serum in parallel with the commercial assay. The in-house assay showed reactive results for the undiluted sample (OD = 0.657) and at a 1:10 dilution (OD = 0.423), whereas the sample was classified as non-reactive at dilutions of 1:100 or greater. The commercial assay showed a comparable reactivity profile, with reactive results for the undiluted and 1:10 diluted samples and non-reactive results from the 1:100 dilution onward. Although differences in OD values were observed between the assays, these differences are expected due to the distinct cut-off values established for each method. Under the conditions evaluated, the in-house ELISA demonstrated an analytical detection limit corresponding to a 1:10 serum dilution, showing comparable dilutional sensitivity to the commercial assay (Table 4).

Batch-to-batch variation was assessed by comparing two independent batches of the recombinant multi-epitope HCV protein (Table 5). Positive (n = 5) and negative (n = 5) serum samples were tested in triplicate for each batch. The mean OD values for the negative controls were 0.119 ± 0.070 for Batch 1 and 0.154 ± 0.082 for Batch 2, corresponding to coefficients of variation (CV) of 59.1% and 53.4%, respectively, with no statistically significant difference between batches (*p* = 0.625). For the positive controls, mean OD values were 0.877 ± 0.149 for Batch 1 and 0.825 ± 0.155 for Batch 2, with CVs of 17.0% and 18.8%, respectively, and no statistically significant difference between batches (*p* = 0.812). These findings indicate consistent assay performance between the two independent antigen batches. The OD value for each sample was calculated as the mean of three technical replicates, and comparisons between batches were performed using the paired Wilcoxon test (Table 4).

The inter-operator reproducibility of the in-house ELISA was evaluated using negative controls (NC) and positive controls (PC) across three experimental days, with three replicate measurements performed by each operator per day. For NC samples, Operator 1 showed a median OD of 0.183 (Q1–Q3: 0.132–0.230), whereas Operator 2 showed a median OD of 0.147 (Q1–Q3: 0.106–0.208). No statistically significant difference was observed between operators (Wilcoxon signed-rank test, *p* = 0.787) (Figure 5).

For PC samples, the median OD was 1.287 (Q1–Q3: 0.930–1.335) for Operator 1 and 1.143 (Q1–Q3: 0.884–1.292) for Operator 2. Similarly, no statistically significant difference was observed between operators (*p* = 0.675). The boxplot distribution showed overlapping OD values between operators for both control groups, supporting the consistency of assay performance across operators. Overall, these findings indicate good inter-operator reproducibility of the in-house ELISA under the evaluated experimental conditions (Figure 5).

## 4. Discussion

In this study, we developed and evaluated an in-house ELISA based on a recombinant HCV multi-epitope protein for the detection of anti-HCV antibodies in serum. The assay demonstrated high diagnostic performance, highlighting its potential for use in diagnostic testing.

The use of a multi-epitopic recombinant protein comprising antigenic regions of HCV structural and non-structural proteins (core, NS3, NS4, NS5A, and NS5B) likely contributed to the strong immunoreactivity observed. Previous studies have demonstrated that multi-epitopic antigens significantly improve diagnostic performance compared to single-antigen approaches, as they increase the likelihood of detecting heterogeneous antibody responses among individuals [13]. Furthermore, a recent systematic review summarizing the performance of recombinant antigens in HCV immunodiagnostic assays reinforced the advantages of multi-epitopic designs, highlighting their ability to improve sensitivity and broaden antibody recognition across different viral genotypes and patient populations [10]. Compared with these previously reported constructs, our antigen was specifically designed to incorporate consensus immunogenic regions derived from HCV genotypes 1a, 1b, and 3a, which are the predominant circulating genotypes in Brazil. In addition, epitope selection combined published immunological evidence with predictions from three independent immunoinformatics tools, followed by multiple sequence alignment to generate consensus sequences. This rational design aimed to increase antigenic coverage while considering the genetic diversity of HCV circulating in the target population.

This is particularly relevant in HCV infection, where the humoral immune response varies depending on the viral genotype, the stage of infection, and individual immunological variability. The inclusion of epitopes derived from genotypes 1a, 1b, and 3a, which are highly prevalent in Brazil, further reinforces the diagnostic applicability of the assay across diverse populations. However, the performance of the recombinant antigen was not evaluated according to HCV genotype, and its applicability in populations where other genotypes predominate remains to be established. In addition, HIV co-infection was identified in a small proportion of anti-HCV reactive individuals (9/127). The influence of immunosuppression on assay sensitivity was not specifically assessed in this study, as it was not part of the original study objectives. Although the small number of HIV co-infected individuals limited further subgroup analysis, no apparent reduction in assay sensitivity was observed among these participants. Future studies including larger and more diverse cohorts, with broader genotype representation and geographically distinct populations, will be important to further evaluate assay performance in different clinical settings, assess the generalizability of this approach, and determine its potential for international application.

Consistent with these findings, previous ELISA-based studies using recombinant multi-epitope constructs have reported high sensitivity and specificity, often comparable to commercial assays [16]. It has also been shown that no single HCV antigen is sufficient to achieve optimal diagnostic accuracy, as different viral proteins contribute variably to antibody recognition. Core and NS3 proteins are associated with strong immunogenic responses, while NS4 and NS5 proteins contribute to improving overall assay coverage. Therefore, the design strategy employed in this study aligns with current advances in HCV serodiagnostics, supporting the high performance observed in serum samples [17].

The assay achieved excellent discriminative capacity (AUC = 0.968), high specificity (98.5%), and almost perfect agreement with the reference method (Kappa = 0.853). These results indicate that the in-house ELISA performs comparably to established commercial assays and is highly reliable for conventional serological diagnosis. Nevertheless, the sensitivity (86.6%) indicates that a subset of anti-HCV-reactive samples identified by the reference commercial ECLIA assay was not detected by the in-house ELISA. This difference may be related to the higher analytical sensitivity of automated ECLIA platforms, which incorporate signal amplification systems and highly optimized assay conditions that generally provide superior detection of low-level antibody reactivity compared with conventional colorimetric ELISA assays. In addition, the diagnostic performance of the in-house assay may be further improved through additional optimization steps, including refinement of antigen coating conditions, serum and conjugate dilutions, assay cut-off determination, and validation using larger and more diverse sample panels.

Additional analytical evaluations supported the consistency of the in-house ELISA under the conditions tested. The detection limit assessment showed that anti-HCV reactivity was maintained up to a final serum dilution of 1:100, with mean OD values remaining above the established cut-off, indicating that the assay retained its ability to detect anti-HCV antibodies after progressive sample dilution. Furthermore, comparison of two independent production batches of the recombinant multi-epitope protein showed consistent assay responses, with batch-to-batch variation assessed through replicate testing of anti-HCV-positive and anti-HCV-negative samples. The evaluation of repeatability and reproducibility further demonstrated consistent assay performance across operators and experimental days. No statistically significant differences were observed between operators for either negative controls (*p* = 0.787) or positive controls (*p* = 0.675), supporting the inter-operator reproducibility of the assay. Together, these findings provide additional evidence of the analytical consistency of the assay and support its further evaluation in larger validation studies.

This study has several limitations that should be considered when interpreting the results. First, all samples were obtained from a biobank, which may introduce selection bias and may not fully represent the heterogeneity of populations encountered in routine clinical settings. Second, although the number of samples included was sufficient to demonstrate the diagnostic potential of the assay, larger studies are needed to provide more precise estimates of sensitivity and specificity. Moreover, because this study was based on retrospectively collected archived serum samples, comprehensive clinical information was not consistently available for all participants. Consequently, variables such as disease stage, liver disease severity, antiviral treatment status, and HIV co-infection could not be systematically assessed. Although HBV status (HBsAg) was available and included in the characterization of the study population, complete HIV serological data were not available for all participants, precluding a reliable evaluation of HIV co-infection. Therefore, the potential influence of these clinical characteristics on assay performance remains to be investigated in future prospective studies with more comprehensive clinical data. In addition, the performance of the in-house ELISA was evaluated using a retrospective sample set under controlled laboratory conditions. Therefore, further studies are required to assess its robustness and reproducibility in prospective and multicenter settings. Finally, external validation using independent sample cohorts was not performed and remains necessary to confirm the generalizability of the assay and its applicability to different epidemiological contexts.

## 5. Conclusions

In conclusion, the recombinant multi-epitope HCV protein was successfully expressed, purified, and applied as the capture antigen in an in-house ELISA for the detection of anti-HCV antibodies. The assay demonstrated excellent diagnostic performance, with high specificity, strong discriminative ability, and almost perfect agreement with established reference methods. The detection limit assessment further demonstrated that the in-house ELISA maintained anti-HCV reactivity up to a final serum dilution of 1:100, showing a comparable detection limit to that observed with the commercial assay.

The incorporation of immunodominant epitopes derived from multiple HCV proteins and genotypes likely contributed to the broad antibody recognition observed, supporting the effectiveness of the antigen design strategy. These findings indicate that the developed ELISA represents a promising and potentially cost-effective approach for serological screening of HCV infection.

Although additional studies involving larger and independent populations and broader analytical validation across different laboratories and clinical settings are required to further validate its performance, the results demonstrate the potential of this recombinant antigen as a promising tool for expanding access to hepatitis C diagnosis and supporting global efforts toward HCV elimination.

## 6. Patents

The findings described in this manuscript are subject to ongoing intellectual property protection efforts. A patent application based on the reported technology is currently being prepared by the authors’ institution. Consequently, certain proprietary details have been omitted to preserve patentability and confidentiality.

## Figures and Tables

**Figure 1:**
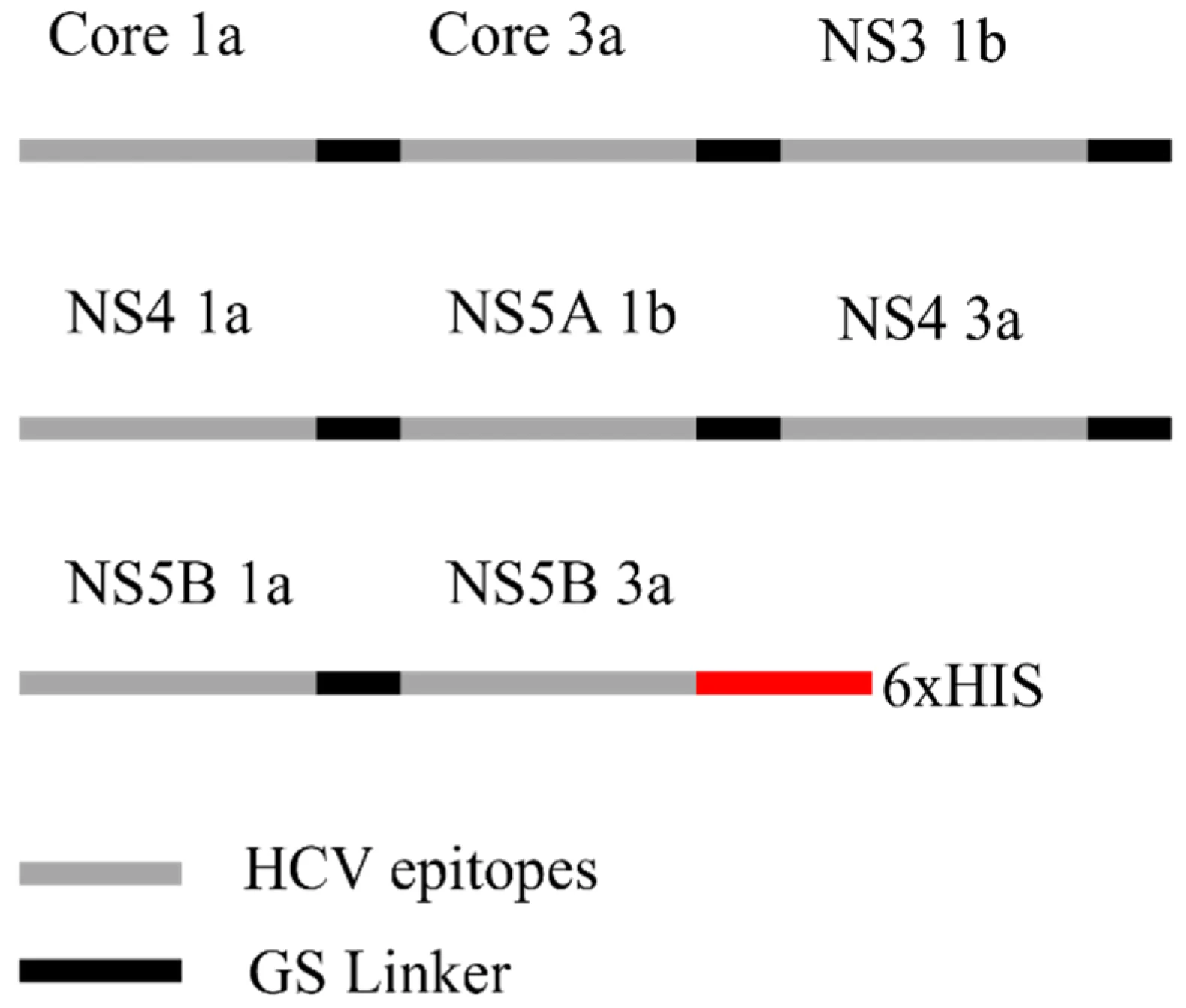
Schematic representation of the recombinant multi-epitope HCV gene construct. Legend: Schematic diagram of the synthetic multi-epitope gene constructed from immunodominant regions of HCV proteins (core, NS3, NS4, NS5A, and NS5B) representative of genotypes 1a, 1b, and 3a. The epitopes were linked using flexible peptide sequences and cloned into the pET21a expression vector, incorporating a C-terminal polyhistidine (His) tag for recombinant protein expression and purification in *Escherichia coli*.

**Figure 2:**
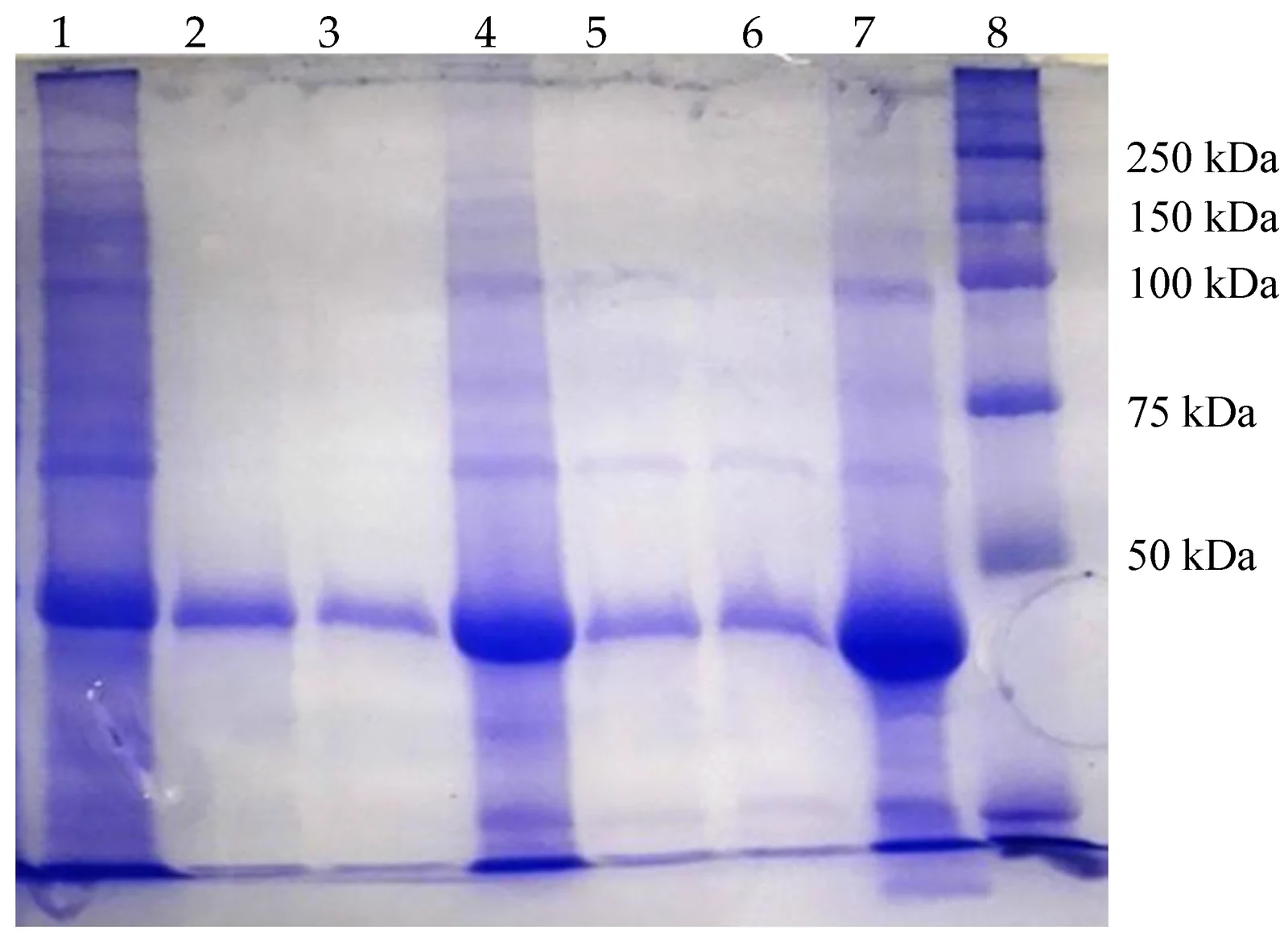
SDS-PAGE analysis of recombinant multi-epitope HCV protein expression and purification. Legend: SDS-PAGE analysis showing expression and purification of the recombinant multi-epitope HCV protein (~43 kDa). Lane 1: crude extract; lanes 2–3: purified fraction 1; lane 4: concentrated fraction 1; lanes 5–6: purified fraction 2; lane 7: concentrated fraction 2; lane 8: molecular weight marker. The enrichment of the target protein band after affinity chromatography indicates successful purification.

**Figure 3:**
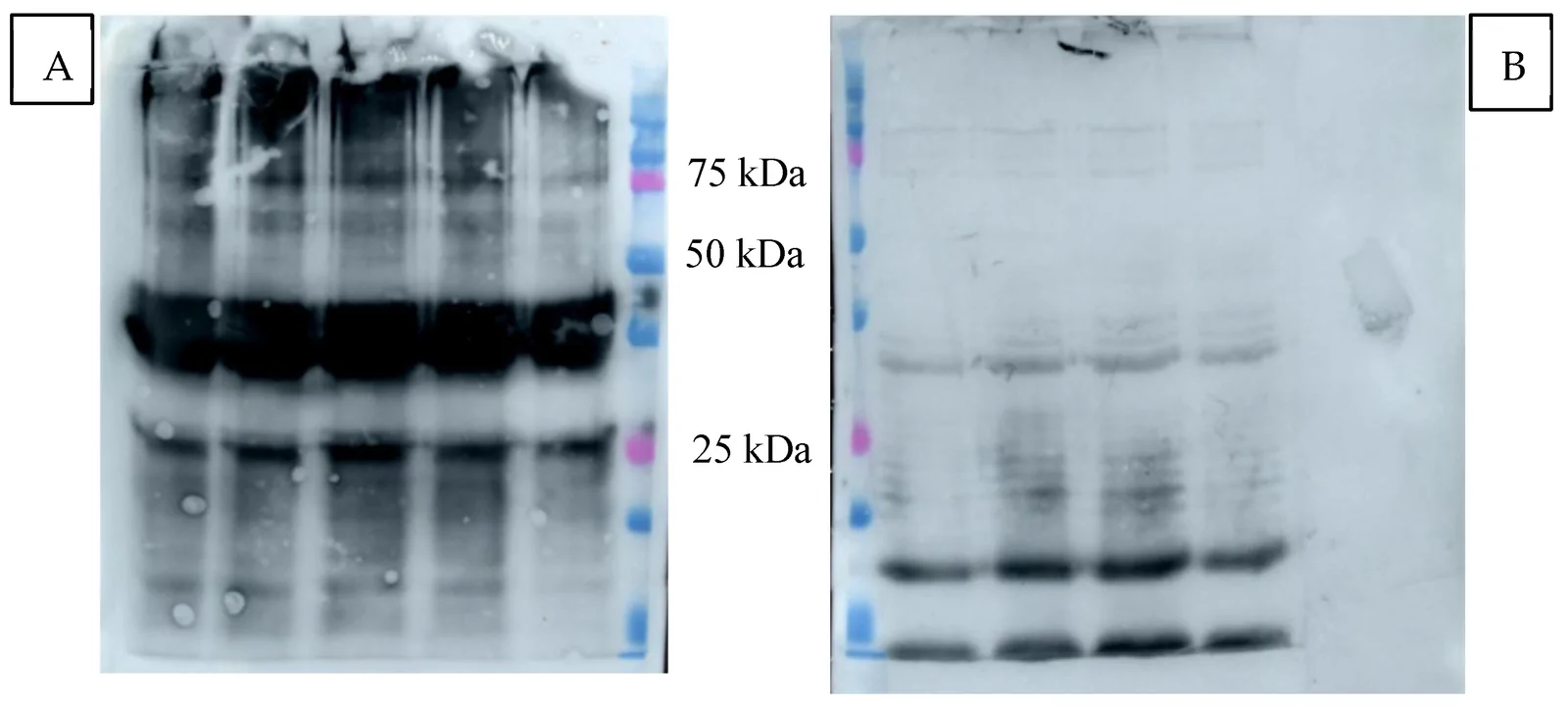
Western blot analysis confirming antigenicity of the recombinant HCV protein. Legend: Western blot analysis demonstrating the antigenic reactivity of the recombinant multi-epitope HCV protein. (**A**) Membrane incubated with anti-HCV reactive serum samples showing specific protein recognition. (**B**) Membrane incubated with non-reactive serum samples. The molecular weight marker used for protein size estimation was the Precision Plus Protein™ Dual Color Standard (Bio-Rad Laboratories, Hercules, CA, USA), with the molecular weight bands indicated by different colors.

**Figure 4:**
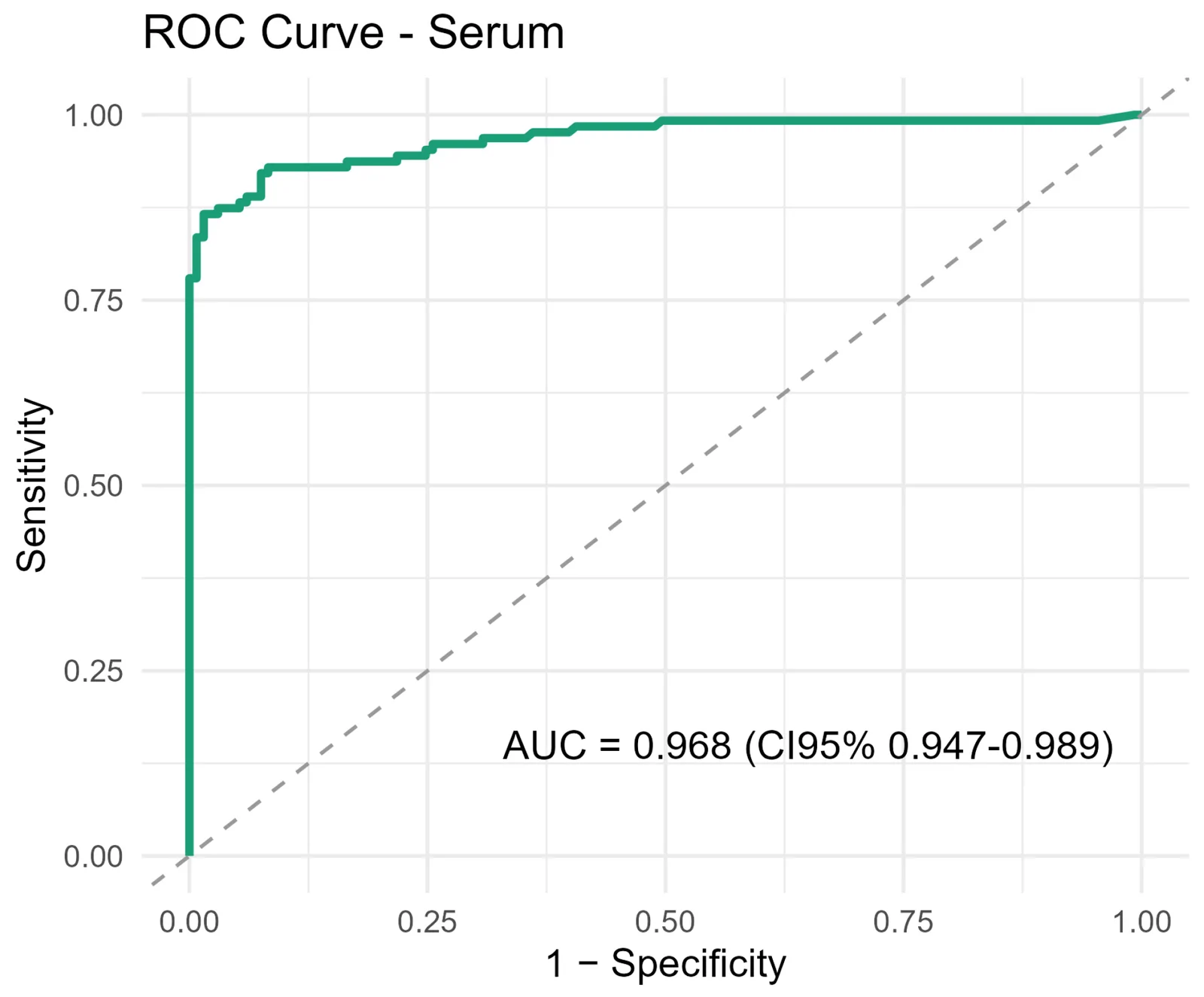
Receiver operating characteristic (ROC) curve for serum samples. Legend: Receiver operating characteristic (ROC) curve evaluating the diagnostic performance of the in-house ELISA using serum samples. The assay demonstrated excellent discriminative ability, with an area under the curve (AUC) of 0.968. The optimal cutoff value was determined using the Youden index. The dashed diagonal line represents the performance expected from a non-discriminatory classifier (AUC = 0.50).

**Figure 5:**
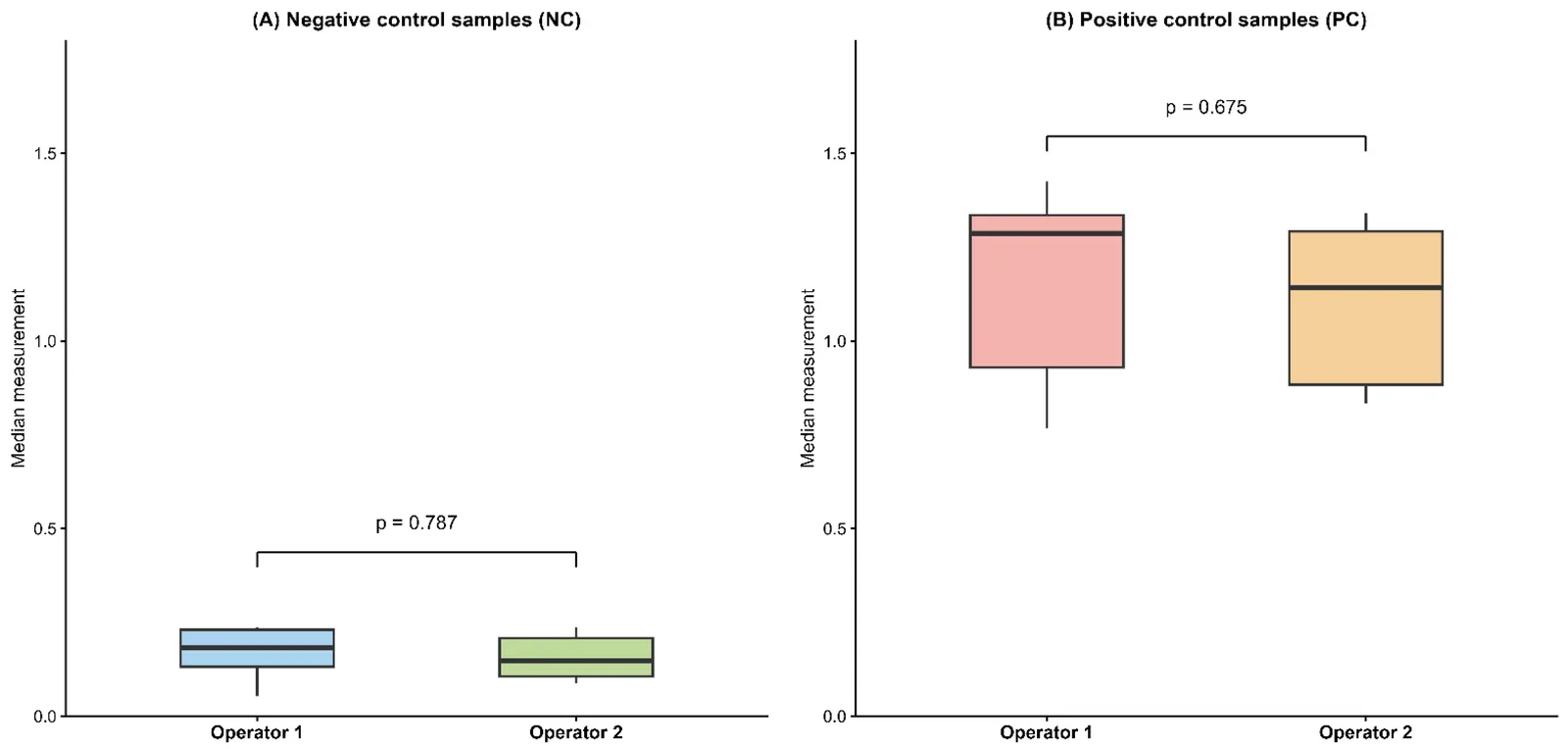
Repeatability and reproducibility of the in-house ELISA.

**Table 1:** Composition of the synthetic multi-epitope HCV gene construct.

Gene/Protein of Origin	Genotype	Position (aa)
Core	1a	1–45
Core	3a	51–95
NS3	1b	101–148
NS4	1a	154–195
NS5a	1b	201–218
NS4	3a	224–265
NS5B	1a	271–299
NS5B	3a	305–333

Legend: Immunogenic regions derived from structural and non-structural hepatitis C virus (HCV) proteins used for the construction of the recombinant multi-epitope gene. The selected peptide sequences represent genotypes 1a, 1b, and 3a and were connected using flexible linkers to form the final synthetic construct.

**Table 2:** Demographic and virological characteristics of the study population.

Characteristic	HCV-Positive Patients (n = 127)	HCV-Negative Patients (n = 133)
Age, Mean ± SD	56, 1 ± 12.1	50, 0 ± 11.7
Male sex at birth, n (%)	50 (39.4)	60 (45.1)
HBsAg positive, n (%)	6 (4.7)	84 (63.2)
HCV-RNA, n (%)	58 (45.7) **	NA *
HCV viral load (log10), Mean ± SD	5.9 ± 0.9 **	NA *

* NA, not applicable. ** Viral load was available only for Anti-HCV positive samples.

**Table 3:** Diagnostic performance and agreement analysis of the in-house ELISA based on the recombinant multi-epitope HCV antigen.

Performance Measure	Value
True positive (n)	110
False positive (n)	2
True negative (n)	131
False negative (n)	17
Sensitivity (%, CI95%)	86.6 (79.6–91.5)
Specificity (%, CI95%)	98.5 (94.7–99.6)
Accuracy (%, CI95%)	92.7 (88.9–95.3)
ROC (AUC, CI95%)	0.968 (0.947–0.989)
Overall Concordance (%, CI95%)	92.7 (88.9–95.3)
Cohen’s Kappa Coefficient (κ, CI95%)	0.853 (0.790–0.916)
Kappa Interpretation	Almost perfect agreement

Legend: Diagnostic performance parameters of the in-house ELISA developed using the recombinant multi-epitope HCV protein. The table summarizes the area under the ROC curve (AUC), sensitivity, specificity, overall accuracy, overall concordance rate, and Cohen’s kappa coefficient obtained by comparison with the reference methods. The kappa coefficient was interpreted according to the Landis and Koch classification, indicating almost perfect agreement between methods.

**Table 4:** Limit of detection of the in-house anti-HCV ELISA compared with a commercial assay.

Dilution Factor	Do In-House	Result	DO Commercial Kit	Result
Pure	0.657	reactive	3.000	reactive
1:10	0.423	reactive	3.000	reactive
1:100	0.159	non reactive	0.677	non reactive
1:1000	0.109	non reactive	0.224	non reactive
1:10,000	0.165	non reactive	0.162	non reactive
1:100,000	0.203	non reactive	0.158	non reactive

**Table 5:** Comparison between Batch 1 and Batch 2.

Sample Group	Batch 1, Mean (SD) (CV%)	Batch 2, Mean (SD) (CV%)	*p*-Value
Negative samples (n = 5)	0.119 (0.070) (59.142)	0.154 (0.082) (53.409)	0.625
Positive samples (n = 5)	0.877 (0.149) (16.958)	0.825 (0.155) (18.806)	0.812

Legend: Mean optical density (OD), standard deviation (SD), and coefficient of variation (CV%) for anti-HCV-negative and anti-HCV-positive serum samples tested in two independent batches of the recombinant multi-epitope HCV antigen. Five samples per group were tested in triplicate for each batch. Batch comparisons were performed using the paired Wilcoxon test.

## Data Availability

The data presented in this study are not publicly available because they contain proprietary information associated with ongoing intellectual property protection and patent application procedures. Data may be made available from the corresponding author upon reasonable request and subject to institutional approval and confidentiality requirements.

## Abbreviations

The following abbreviations are used in this manuscript:

Abbreviation	Definition
AUC	Area Under the Curve
BSA	Bovine Serum Albumin
CI	Confidence Interval
DAAs	Direct-Acting Antivirals
DNA	Deoxyribonucleic Acid
ECLIA	Electrochemiluminescence Immunoassay
ELISA	Enzyme-Linked Immunosorbent Assay
GHSS	Global Health Sector Strategies
HCC	Hepatocellular Carcinoma
HCV	Hepatitis C Virus
IEDB	Immune Epitope Database
IMAC	Immobilized Metal Affinity Chromatography
IPTG	Isopropyl β-D-1-thiogalactopyranoside
kDa	Kilodalton
NAT	Nucleic Acid Test
Ni-NTA	Nickel-Nitrilotriacetic Acid
NS3	Non-Structural Protein 3
NS4	Non-Structural Protein 4
NS5A	Non-Structural Protein 5A
NS5B	Non-Structural Protein 5B
OD	Optical Density
PBS	Phosphate-Buffered Saline
PBS-BSA	Phosphate-Buffered Saline Containing Bovine Serum Albumin
PBST	Phosphate-Buffered Saline Containing Tween 20
PCR	Polymerase Chain Reaction
RNA	Ribonucleic Acid
ROC	Receiver Operating Characteristic
RT-PCR	Reverse Transcription Polymerase Chain Reaction
SDS-PAGE	Sodium Dodecyl Sulfate–Polyacrylamide Gel Electrophoresis
TBST	Tris-Buffered Saline with Tween 20
TMB	Tetramethylbenzidine
WHO	World Health Organization
